# Serum melatonin levels in type 2 diabetic patients with depressive symptoms compared to non-depressed individuals

**DOI:** 10.22088/cjim.15.3.421

**Published:** 2024-08-01

**Authors:** Neda Kazemipoor, Alireza Arefzadeh, Davood Dalil, Maryam Shiehmorteza, Seyyed Mohammad Hosseini

**Affiliations:** 1 Department of Clinical Pharmacy, Faculty of Pharmacy, Tehran Medical Sciences Islamic Azad University, Tehran, Iran; 2 Department of Endocrinology, Farhikhtegan Hospital, Faculty of Medicine, Tehran Medical Sciences Islamic Azad University, Tehran, Iran; 3 Student Research Committee, Faculty of Medicine, Shahed University, Tehran, Iran; 4 Department of Clinical Pharmacy, Pharmaceutical Sciences Branch, Islamic Azad University, Tehran, Iran

**Keywords:** Melatonin, Diabetes mellitus type 2, Depression, Depressive diabetic patients, Circadian rhythm, Metabolism

## Abstract

**Background:**

Melatonin, mainly regulating the body's circadian rhythm, may have protective effects against type 2 diabetes mellitus (DM2)-induced depression due to its antioxidant and regulatory impact in the pathogenesis of both DM2 and depression. This study aimed to find the association of serum melatonin levels with depression in DM2 patients.

**Methods:**

A total of 50 DM2 patients were recruited in this retrospective cross-sectional study and divided into 25 patients with depression (DM2-DP) and 25 without depression symptoms (DM2-NDP). Depression was diagnosed using the Hospital Anxiety and Depression Scale (HADS) assessment. Fasting blood samples were collected and examined for the level of serum melatonin and other biomarkers. All statistical analysis was performed by SPSS software Version 22, and a p-value less than 0.05 was considered statistically significant for all tests.

**Results:**

The depression score was significantly lower in DM2-NDP than DM2-DP (p< 0.001). The mean weight was significantly lower in the DM2-DP group (P= 0.021). Total cholesterol, triglyceride, and anxiety scores were higher, and the melatonin level was lower in DM2-DP. The correlation of melatonin levels was positive with age, DBP, HbA1C, FBS, and TG. In contrast, it was negative with male gender, BMI, diabetes duration, SBP, TC, family history of DM, depression score, and anxiety score. However, no significant differences were seen.

**Conclusion:**

Lower melatonin may be associated with depression and anxiety in patients with DM2. The serum melatonin level might be a strong predictor of depression in DM2 patients.

Diabetes mellitus (DM) is a common metabolic disorder that is a growing health challenge worldwide due to its increasing prevalence, chronic complications, and body organ dysfunction. DM is identified by the abnormally excessive blood glucose level as a result of inadequate insulin secretion, called type 1 DM (DM1), or insulin resistance, called type 2 DM (DM2). The clinical burden of diabetes control may increase the risk of depression and brain function changes in patients (1). 

Depression is a serious mental disorder that may cause emotional and physical issues such as feeling sadness, disinterest in activities once enjoyed, weight loss or gain, insomnia, etc. (2, 3). Epidemiologic studies have shown that diabetic patients often suffer from depression, at least twice as high as those in non-diabetic populations (4). It is reported that 43 million people with diabetes worldwide have symptoms of depression (5).

Diabetes combined with depression leads the patient to a clinical condition with a poorer prognosis, in which the outcomes including quality of life, complications, and life expectancy are weaker compared to having each disease alone (6). Melatonin is a neuroendocrine hormone that the pineal gland secretes to regulate the circadian rhythm (7). It can effectively modulate multiple organ functions and acts as an anti-stress agent (8). Any disturbance of melatonin level can lead to metabolic disorders such as DM2, and also depression (9). Previous studies demonstrated the controversial relationship between melatonin and depression. Although earlier studies showed lower melatonin levels in depressed people, recent studies have observed a higher level of melatonin or at least the same level in depressed individuals compared with healthy ones. Carvalho et al. demonstrated that the patients with depression have a similar melatonin levels with healthy individuals (10). Kor et al., in a study of DM1 patients, and Peschke et al., by studying DM2 patients, found that both DM1 and DM2 patients displayed significantly lower levels of serum melatonin (11, 12). Meng et al. suggested that melatonin regulates the circadian insulin secretion by the pancreatic Langerhans islets (13). Thus, melatonin may have a role in the development of diabetes due to altering the phase of insulin secretion, which is an essential characteristic of DM2 (14). Also, oxidative stress (OXS) increases in diabetic patients which plays an important role in causing various complications, including depression (15). Moreover, the melatonin easily passes the blood-brain barrier and scavenges free radicals, leading to better antioxidant activity and decreasing the amount of reactive oxygen species (ROS) (16). 

Therefore, melatonin may have significant protective effects against anxiety-like behaviors and DM2-induced depression due to its antioxidant and regulatory effect in the pathogenesis of both DM2 and depression. However, the relationship between melatonin and depression in patients with DM2 is yet to be identified. Thus, the aim of this study was to find the association of serum melatonin levels with depression in type 2 diabetic patients.

## Methods

**Study population:** The present cross-sectional study was conducted on all type 2 diabetic patients on oral medication presented to the endocrinology departments of BooAli and Amir-al-Momenin Hospitals, Tehran, Iran, who met the inclusion criteria. Based on a study by Khaleghipour et al. (17) and using the sample size formula to compare the mean of two independent samples (calculation is provided as supplementary materials), a total of 50 DM2 patients (25 patients in each group) was engaged in this study with a history of DM2 for more than one year. Diabetes was diagnosed according to the World Health Organization 1999 (18). Family history of type 2 diabetes, age over 35 years, negativity of diabetes autoantibodies, and no history of autoimmune disease were among the factors that were taken into consideration in the diagnosis of type 2 diabetes.

Depression was diagnosed based on the Hospital Anxiety and Depression Scale (HADS) assessment. All included patients completed the HADS questionnaire. Based on the HADS score, they were classified into two groups: DM2 patients with depressive symptoms (DM2-DP) and DM2 patients without depressive symptoms (DM2-NDP).

The conditions leading to exclusion of participants were as follows: 1) Patients with DM1. 2) DM2 patients on insulin. 3) Presence of underlying neuropsychiatric disorders. 4) Pregnancy and lactation. 5) Cancer and autoimmune diseases. 6) Taking antidepressants (at least two weeks before the study). 7) Shift workers or recently having intercontinental travel.

**Clinical and laboratory data collection:** Demographic data, including gender and age, and clinical characteristics, including weight, height, body mass index (BMI), systolic and diastolic blood pressure, diabetes history, and family history of diabetes were collected. Laboratory data, including fasting blood sugar (FBS), triglyceride (TG), glycosylated hemoglobin (HbA1c), and total cholesterol (TC), were extracted from the patients’ medical records. Fasting blood samples were collected and centrifuged to separate serum, frozen at −70°C until analysis. We used the enzyme-linked immunosorbent assay (ELISA) to determine the melatonin levels. 

**Hospital anxiety and depression scale (HADS):** The Hospital Anxiety and Depression Scale (HADS) questionnaire contains seven items each for depression and anxiety subscales. Item scoring is based on a four-point scale, from zero to three. The maximum score of each subscale is 21. A 'normal' case is defined based on a total score of 0-7, while scores of 8-10 indicate 'borderline' and 11-21 determine an 'abnormal' case of depression or anxiety. The validity and reliability of the Iranian version of HADS were confirmed before (19).

**Statistical analysis:** The SPSS software Version 22 (SPSS Inc., Chicago, Illinois, USA) was used for statistical analysis. The collected data were reported as mean ± standard deviation (SD) for quantitative variables and as number (percentage) for qualitative ones. The independent two-sample t-test was used to analyze the normally distributed quantitative variables, and the Mann–Whitney U test was used for non-normal data. The chi-square test was applied for the analysis of the qualitative data. A statistically significant difference was defined as a p-value less than 0.05.

**Ethical approval:** This study was conducted ethically in accordance with the World Medical Association Declaration of Helsinki. The Research Ethics Committees of Islamic Azad Tehran Medical Sciences University approved all procedures performed in the current study with the approval number: IR.IAU.PS.REC.1398.177. Written informed consent was obtained from the patients.

## Results

**Figure 1:**
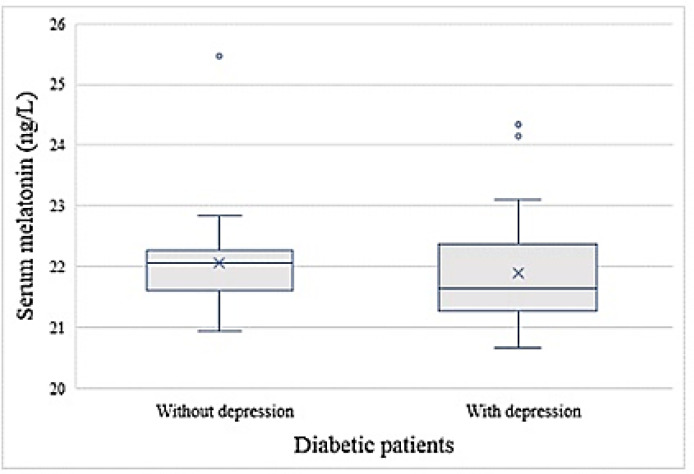
Medians and interquartile ranges of serum melatonin in DM2 patients with and without depressive symptoms

A total of 50 patients with DM2 was enrolled in this study; 25 patients in DM2-DP group and 25 patients in DM2-NDP group. The mean ± SD age of all patients was 52.12 ± 7.36 years with the minimum and maximum of 35 and 60 years old. The other demographic data of the study population is reported in table 1. The results showed no significant difference between the depressive and not depressive groups regarding the gender and family history of DM. Depression score was significantly lower in DM2-NDP than in DM2-DP (p< 0.001). The mean weight of diabetic patients with and without depression were 72.32±12.02 and 82.36±17.25 Kg, respectively. The comparison revealed a significantly lower mean weight in the DM2-DP group (P= 0.021). TC, TG, and anxiety scores were higher, and the melatonin level was lower in DM2-DP (figure 1). However, no significant differences were seen. Also, the differences between the two groups regarding other variables were not statistically significant (p> 0.05).

The comparison of serum melatonin levels in the study population based on demographic and clinical data of patients is demonstrated in table 2. Although the melatonin level was lower in DM2-DP group regarding some characteristics including age <= 50, male gender, BMI, blood pressure > 120/80, HbA1C <= 7.5, and FBS > 130, there was no significant difference between two groups (p > 0.05). 

**Table 1:** Comparison of demographic and clinical characteristics of the study population based on the presence or absence of depressive symptoms (Mean ± SD)

**Variables**	**All** **(n=50)**	**DM2-DP** **(n=25)**	**DM2-NDP (n=25)**	**Z or χ2**	**P -value**
**Age (year)**	52.12 ± 7.36	51.40 ± 8.16	52.84 ± 6.57	- 0.687	0.495
**Male (n (%))**	33 (66)	7 (28)	10 (40)	0.802	0.370
**Height (cm)**	166.08 ± 10.64	163.88 ± 9.24	168.28 ± 11.66	- 1.479	0.146
**Weight (Kg)**	77.34 ± 15.56	72.32 ± 12.02	82.36 ± 17.25	- 2.388	**0.021**
**BMI (Kg/m** ^2^ **)**	27.94 ± 4.30	26.98 ± 4.16	28.92 ± 4.31	- 1.617	0.112
**Diabetes duration (years)**	7.28 ± 6.04	7.20 ± 5.92	7.36 ± 6.28	- 0.093	0.927
**SBP (mmHg)**	128.78 ± 15.76	129.36 ± 18.95	128.20 ± 12.15	0.258	0.798
**DBP (mmHg)**	84.76 ± 12.81	83.32 ± 12.88	86.20 ± 12.85	- 0.791	0.433
**Melatonin (ng/L)**	21.98 ± 0.91	21.90 ± 0.97	22.06 ± 0.85	- 0.626	0.534
**HbA1C (%)**	8.41 ± 2.01	8.36 ± 1.90	8.46 ± 2.14	- 0.171	0.865
**FBS (mg/dL)**	171.56 ± 73.64	166.01 ± 67.12	177.12 ± 80.64	- 0.530	0.599
**TG (mg/dL)**	168.07 ± 80.80	172.57 ± 100.37	163.56 ± 56.68	0.391	0.698
**TC (mg/dL)**	161.99 ± 36.53	164.48 ± 32.36	159.50 ± 40.88	0.478	0.653
**Family history of DM (n (%))**	15 (30)	16 (64)	19 (76)	0.857	0.355
**Depression score**	9.10 ± 4.56	13.16 ± 1.99	5.04 ± 2.05	14.195	**< 0.001**
**Anxiety score**	9.34 ± 5.08	10.72 ± 4.77	7.96 ± 5.10	1.987	0.054


**Table 2:** Comparison of mean melatonin levels (ng/L) in DM2 patients with and without depression symptoms based on demographic and clinical characteristics of patients

**Variables**	**DM2-DP ** **(n=25)**	**DM2-NDP (n=25)**	**Z or χ2**	**P-value**
**Age (year)**				
**<= 50**	21.66 ± 0.015	22.40 ± 1.19	- 1.525	0.147
**> 50**	22.04 ± 1.04	21.87 ± 0.54	0.561	0.579
**Sex (n)**				
**Male**	21.75 ± 0.73	22.35 ± 1.22	- 1.156	0.266
**Female**	21.96 ± 1.07	21.87 ± 0.41	0.322	0.750
**BMI (Kg/m** ^2^ **)**				
**<= 25**	22.29 ± 1.20	22.32 ± 0.36	- 0.057	0.956
**> 25**	21.72 ± 0.83	22.01± 0.90	- 1.042	0.304
**SBP (mmHg)**				
**<= 120**	21.98 ± 1.27	21.91 ± 0.39	0.187	0.855
**> 120**	21.84 ± 0.71	22.19 ± 1.08	- 1.006	0.323
**DBP (mmHg)**				
**<= 80**	22.01 ± 1.13	21.88 ± 0.44	0.428	0.673
**> 80**	21.64 ± 0.33	22.27 ± 1.12	- 1.413	0.176
**HbA1C (%)**				
**<= 7.5**	21.66 ± 1.07	22.11 ± 1.33	- 0.827	0.419
**> 7.5**	22.06 ± 0.91	22.03 ± 0.30	0.113	0.912
**FBS (mg/dL)**				
**<= 130**	22.03 ± 1.23	21.59 ± 0.49	0.935	0.374
**> 130**	21.84 ± 0.87	22.28 ± 0.90	- 1.461	0.154
**Family history of DM (n)**				
**Yes**	22.05 ± 0.92	22.17 ± 0.94	- 0.379	0.707
**No**	21.64 ± 1.07	21.74 ± 0.29	- 0.205	0.841

n, Number; DM2-DP, Type 2 diabetes mellitus patients with depressive symptoms; DM2-NDP, Type 2 diabetes mellitus patients without depressive symptoms; BMI, Body mass index; SBP, Systolic blood pressure; DBP, Diastolic blood pressure; HbA1C, Glycosylated hemoglobin; FBS, Fasting blood sugar; DM, Diabetes mellitus.

The correlation of serum melatonin level with other variables was evaluated by Pearson's correlation (Table 3). The correlation of melatonin levels was positive with age, DBP, HbA1C, FBS, and TG, whereas it was negative with male gender, BMI, diabetes duration, SBP, TC, family history of DM, depression score, and anxiety score. However, there was no significant correlation between serum melatonin with any other indicators (p> 0.05).

**Table 3:** Correlations of serum melatonin level with other variables in T2DM patients

**Variables**	**Melatonin**	
	**r**	**P-value**
**Age (year)**	0.15	0.920
**Male**	- 0.096	0.508
**BMI (Kg/m** ^2^ **)**	- 0.099	0.493
**Diabetes duration (years)**	- 0.809	0.538
**SBP (mmHg)**	- 0.046	0.753
**DBP (mmHg)**	0.071	0.624
**HbA1C (%)**	0.069	0.635
**FBS (mg/dL)**	0.065	0.652
**TG (mg/dL)**	0.110	0.448
**TC (mg/dL)**	- 0.003	0.986
**Family history of DM**	- 0.220	0.124
**Depression score**	- 0.064	0.658
**Anxiety score**	- 0.071	0.624

BMI, Body mass index; SBP, Systolic blood pressure; DBP, Diastolic blood pressure; HbA1C, Glycosylated hemoglobin; FBS, Fasting blood sugar; TG, Triglyceride; TC, Total cholesterol; DM, Diabetes mellitus.

## Discussion

The comorbidity of diabetes and depression is an important clinical challenge because these conditions co-occur frequently, and the outcomes of both diseases are related and can mutually worsen. Studies have argued both diseases are associated with different underlying biologic and behavioral conditions, including activation of the hypothalamic-pituitary-adrenal axis, inflammation, physical inactivity, sleep disorders, poor eating habits, and genetic and environmental risk factors. The diagnosis of this co-occurrence is important in patient's management. Unfortunately, despite the existence of efficient screening tools, depression in diabetics is often not diagnosed. Moreover, the treatments prescribed for depression symptoms in diabetic patients should be selected meticulously. Although medications like antidepressants, as well as neuropsychological interventions, are used to treat the different severity of depression in diabetic patients, they may disrupt blood glucose control (1).

Despite its clinical importance, the pathophysiology of diabetes and depression comorbidity is still unclear. Studies reported OXS may be crucial in the pathology of both diseases due to the increased production of ROS. In chronic major depression, a disruption has been found in the antioxidant mechanism due to increased ROS and decreased activity of antioxidant enzymes. The increase of OXS may also play a key role in many pathological mechanisms of diabetes, such as cell component injury, disturbance of antioxidant enzymes, and disorders of lipid, protein, and DNA metabolisms (20). 

Previous studies have demonstrated various effects of melatonin, including reducing the production of glucose, improving pathways of inflammatory response, and OXS reduction in diabetic patients. Melatonin treatment improved diabetes in experimental models by regulating the metabolism of body lipid and glucose, decreasing the cell resistance to insulin which causes more insulin sensitivity (21). A meta-analysis by Li et al. concluded that melatonin could be an adjuvant therapy for metabolic disorders by reducing hyperinsulinemia and insulin resistance and increasing insulin sensitivity (22). In contrast, a randomized control trial (RCT) by Lauritzen et al. revealed that 3 months treatment with 10 mg of melatonin in male DM2 patients decreases the insulin sensitivity, which is the fundamental in pathophysiology of DM2 (23). Yapislar et al. demonstrated the anti-apoptotic effects of melatonin in the various tissues of rats with DM2, mediated partially by the melatonin MT2 receptor (24). In another *in-vivo* study, they showed that the inflammatory markers IL-1β, IL-6, TNF-α, and NF-κB expressed lower in the liver and adipose tissues of DM2 rats treated with melatonin compared with DM2 rats without melatonin treatment (25). In a meta-analysis of 13,752 Chinese participants, Li et al. found that the polymorphism of melatonin receptor 1B (MTNR1B) gene was significantly associated with DM2 (26). Recently, Amin et al. investigated the role of another melatonin receptor, MTNR1A, in developing DM2 in 212 Italian families and they found that 3 novel MTNR1A genes are remarkably linked to DM2 (27). 

The results of melatonin administration in rats showed improvement of depression and metabolic disorders caused by neuroinflammation, through the downregulation of inflammatory pathways (28). Rebai et al. demonstrated that melatonin reduced the OXS in the prefrontal and hippocampal cortices and by this mechanism, melatonin and fluoxetine can improve depression in diabetic rats (29). An RCT by Seatung et al. demonstrated that the depression symptoms in DM2 patients are more observed in the evening (30). It has been proven that melatonin plays a key role in the initiation and sustaining of sleep. Circadian rhythm changes and sleep disorders have been observed in patients with depression. Zhao et al. investigated the pineal gland abnormality in 50 patients with major depressive disorder and 35 healthy individuals, which revealed that cysts and reduced pineal parenchymal volume are more common in the depressed population compared to the healthy group (31). Furthermore, a new medicine, agomelatine, improves the symptoms of major depression by activating melatonin receptors type I and II and blocking serotonin receptors type II (32). However, a study of 4555 Finnish population without a history of DM revealed that although depression symptoms and the melatonin receptor coding gene expression (rs10830963) independently worsen the glucose profile, there was no significant correlation between the gene expression and depression symptoms or DM2 (9).

Regarding the mentioned effects of melatonin in diabetes and depression and the presence of controversies, this cross-sectional study investigated the relationship between serum melatonin levels and depression in 50 DM2 patients. Depression in DM2 patients was diagnosed based on HADS assessment, which showed that the depression scores in diabetic patients with depressive symptoms were significantly higher than in another group. The mean levels of serum melatonin were lower in the DM2-DP group than in DM2-NDP patients. However, no significant difference was seen. The Pearson correlation demonstrated that serum melatonin levels had a negative correlation with depression and anxiety scores, but were not statistically significant. 

Here, we exclude the DM2 patients who took antidepressant drugs like tricyclic antidepressants (TCAs) or selective serotonin reuptake inhibitors (SSRIs) due to affecting the amount of serotonin (the precursor of melatonin) which may affect the synthesis of melatonin. Thus, the depressive patients were included in the DM2-DP group based on their mood and the HADS depression score. It is possible that other factors except for depression diseases, such as social and familial problems, the burden of diabetes, and poor economic condition, resulted in a high score of depression when answering the questions of HADS assessment, making them DM2-DP patients. Therefore, due to having no history of visiting a psychiatrist or taking antidepressants, there is a hypothesis that the depression of the patients was not of a severe type that disrupted their life, and thus there was no significant change in the melatonin levels of the depressed participants.

A comparison of melatonin levels based on HbA1c level and history of diabetes in patients did not show significant results. The anxiety score was reported high even in non-depressed people. In total, 45% of all samples included patients with anxiety symptoms. However, no significant difference was seen between the melatonin concentration and patients' anxiety scores. Due to the novelty of the subject of this study, most of the previous papers related to this subject are in-vivo studies on animal models. Rebai et al. found that melatonin could suggest as an alternative treatment for depression in diabetic rats owing to showing antidepressant-like and anxiolytic effects (33). In 2019, Ergenc et al. evaluated depression and anxiety in streptozocin-induced diabetic rats. The results revealed that melatonin administration could have antidepressant and anti-anxiety effects in diabetic rats by regulating mediators' secretion and reducing OXS in the prefrontal cortex and hippocampus (34). Rahman et al. demonstrated that a combination of oral melatonin and exercise improves the anxiety and depression-like behaviors in the rat model of DM2 by reducing the levels of serum corticosterone and inflammatory cytokines in hippocampus tissue (35). There are previous studies which reported conflicting results investigating melatonin levels in depressed diabetic patients, current study correlates with studies that showed no significant change in melatonin in depression (10, 36-39). An RCT of 116 patients with DM2 showed that administration of agomelatine for 12 weeks resulted in lower depression scores than paroxetine. Interestingly, the agomelatine-treated group had a remarkable reduction in HbA1c levels compared to the paroxetine-treated group (40). Ostadmohammadi et al. investigated the effects of 12 weeks of melatonin supplementation on diabetic hemodialysis patients in an RCT and found that melatonin significantly reduces the depression index of the patients (41). Zhang et al. showed that lower serum melatonin levels are significantly associated with mild cognitive impairment (42).There were several limitations to our study. First, as we used the HADS as the only assessment tool to evaluate depression in DM2 patients, a more depression score should be used to obtain more accurate results. Second, all blood samples were taken from 8 to 11 A.M. It should be better than samples that were taken twice in the morning and at night, due to changes in melatonin concentration associated with the amount of light. Third, conducting a study including a larger population and considering the group of depressed patients without diabetes and healthy people can lead to more accurate and reliable results. Fourthly, this is a cross-sectional study, and longitudinal interventional studies will be required to evaluate the effects of melatonin treatment in diabetic patients with and without depression and comparing with a control group.In conclusion, melatonin level declined in the DM2-DP and was negatively associated with depression and anxiety, but not statistically significant. The serum melatonin level might be a strong predictor of depression in DM2 patients. However, more studies investigating this subject are required to clarify this association.
